# The Prognostic Impact of the Tumor Immune Microenvironment in Synovial Sarcoma: An Immunohistochemical Analysis Using Digital Pathology and Conventional Interpretation

**DOI:** 10.3390/jpm15050169

**Published:** 2025-04-25

**Authors:** Emilio Medina-Ceballos, Francisco Giner, Isidro Machado, Begoña Heras-Morán, Mónica Espino, Samuel Navarro, Antonio Llombart-Bosch

**Affiliations:** 1 Pathology Department, Hospital Clínico Universitario de Valencia, 46010 Valencia, Spain; emilio.medinacs@gmail.com (E.M.-C.); heras_beg@gva.es (B.H.-M.); mespino@fivo.org (M.E.); samuel.navarro@uv.es (S.N.); 2 Pathology Department, University of Valencia, 46010 Valencia, Spain; francisco.giner@uv.es (F.G.); antonio.llombart@uv.es (A.L.-B.); 3 Pathology Department, University Hospital La Fe, 46010 Valencia, Spain; 4 Pathology Department, Instituto Valenciano de Oncología, 46009 Valencia, Spain; 5 Patologika Laboratory, Quirón-Salud, 46010 Valencia, Spain; 6 Cancer CIBER (CIBERONC), 28029 Madrid, Spain

**Keywords:** synovial sarcoma, tumor microenvironment, immune microenvironment, tumor-infiltrating lymphocytes, digital pathology, tumor-associated macrophages

## Abstract

Background and Objectives: Innate and adaptive immune responses serve a crucial role in neoplasms. The interaction of immune cells with the neoplastic tissue influences tumor behavior, resulting in either pro-tumorigenic or anti-tumorigenic effects. However, the prognostic significance of the tumor immune microenvironment (TIME) in synovial sarcoma (SS) remains poorly studied. This study aimed to analyze the TIME of SS to determine its impact on the prognosis by examining the intratumoral lymphocytic and macrophagic infiltrate and its potential correlation with survival and recurrence. Methods: We conducted a retrospective observational study of 49 fusion-confirmed SS cases collected from two different institutions. We obtained clinical and follow-up data, and SSs were histologically classified according to WHO criteria. Immunohistochemical analysis, including of CD163, CD68, CD3, CD8, and CD20, was conducted in tissue microarrays using an analog scale. We examined the whole-slide tissue for the 23 cases with sufficient material available and then assessed the positive area by scanning the slides and analyzing the images using QuPath (0.4.4, Belfast, Northern Ireland) to calculate the positive area in an immune hotspot. We correlated the expression of these markers with clinical outcomes. A log-rank test and Kaplan–Meyer curves were used as appropriate (significance: *p* ≤ 0.05). Results: The most frequent morphological subtype was monophasic (59.6%), followed by biphasic (26.9%) and undifferentiated (7%). The mean disease specific survival (DSS) was 55.3 months, with a median of 33 months. The median overall survival (OS) was 50 months (range: 2–336 months). Both evaluation methods showed a good correlation for all antibodies, with Chi-square values of *p* < 0.05. All cases showed variable amounts of CD163-positive macrophages. The cases that showed a higher density of CD163-positive macrophages in whole-slide images subjected to digital analysis demonstrated an improved OS and DSS on Kaplan–Meier curves. Cases with lower CD8 and CD3 positivity showed a tendency toward faster progression and a slightly worse prognosis. Conclusions: The tumor immune microenvironment in sarcomas is a complex system that requires further investigation to fully understand its impact on tumorigenesis and patient clinical outcomes. Our results demonstrate that a higher amount of intratumoral CD163-positive macrophage infiltrate is associated with an increased OS and DSS. Our findings show that digital pathology is more precise than subjective quantitative analysis.

## 1. Introduction

Synovial sarcoma (SS) is a rare and aggressive soft tissue malignancy, accounting for 5–10% of all soft tissue sarcomas. It is defined by a characteristic t(X;18)(p11;q11) translocation, which generates the *SS18::SSX1/2/4* fusion gene [1,2]. Despite its name, SS rarely involves synovial tissue and predominantly arises in para-articular regions, especially near large joints like the knee. Most cases occur in the deep soft tissue of the extremities (70%), with a greater tendency for the lower limbs, while it appears less frequently in the head and neck (5–10%) and trunk (15%) areas and rarely in visceral locations [3]. The exact origin of this tumor remains uncertain; although recent studies have suggested a potential neural origin due to TRPA1 expression, it continues to be categorized as a sarcoma of uncertain differentiation [4].

The cornerstone of SS treatment is surgical excision with negative margins, often combined with radiotherapy or chemotherapy depending on the tumor size and patient factors such as age. The prognosis of SS still remains poor, and despite progress in surgery, radiotherapy, and chemotherapy over recent decades, the outcomes have shown little improvement. Advances in imaging and adjuvant therapies have allowed limb salvage surgery to largely replace the historical reliance on amputation. The long-term overall survival rates are 80%, 70%, and 56% at 5, 10, and 20 years, respectively, with the tumor size and negative margins being the main prognostic factors [5]. SS patients who relapse have been reported to have only a 30% 5-year survival rate. Current recommendations for complete resection (R0) suggest a 1.0 cm margin for soft tissue sarcomas (National Comprehensive Cancer Network) or a 0.5 cm margin, recommended by the Children’s Oncology Group [6,7]. While chemotherapy is commonly employed, it has not consistently demonstrated improved survival, particularly in older patients and cases involving larger or more aggressive tumors [2,8].

The tumor immune microenvironment (TIME) plays a critical role in modulating the tumor progression, therapeutic response, and patient prognosis across various cancer types. It encompasses a complex network of immune cells, stromal components, signaling molecules, and vasculature that interact dynamically with tumor cells. In sarcomas, including synovial sarcoma, the TIME has emerged as a key area of interest, particularly given the historically limited immunogenicity of those tumors regarded as “cold tumors” [9]. Nonetheless, the study of the TIME in soft tissue tumors has shown potential prognostic relevance and may inform future therapeutic strategies [10]. Understanding the type of immune infiltrate and its spatial organization may offer valuable insights into the mechanisms of immune evasion and identify opportunities for targeted therapies. This study aimed to analyze the TIME of SS to determine its impact on the prognosis by examining the intratumoral lymphocytic and macrophagic infiltrate and its potential correlation with survival and recurrence.

## 2. Materials and Methods

Forty-nine fusion-confirmed synovial sarcomas (SSs) diagnosed between 2006 and 2023 were retrospectively collected from the pathology departments of the Hospital Clínic Universitari, Valencia, and the Hospital Universitari i Politècnic La Fe, Valencia. Formalin-fixed, paraffin-embedded tissue samples were retrieved along with their corresponding hematoxylin and eosin (H&E) slides for histological evaluation. The cases included in this study were part of a previously curated series of SSs [11], selected based on the availability of representative tumor material suitable for tissue microarray (TMA) construction and the presence of clinical follow-up data. All the included tumors had been molecularly confirmed through the detection of SS18 rearrangement. The exclusion criteria for the overall cohort were the absence of adequate tumor tissue for TMA analysis or a lack of available clinical or follow-up data.

A subset of 23 tumors from this series was selected for whole-slide immunohistochemical analysis. This subgroup was based on the availability of sufficient and well-preserved tumor material at the time of digital analysis, which took place several months after the initial curation of the cohort. Cases were excluded from this portion of the study if the tissue had been depleted in prior research, was fragmented, or had been required for patient care.

The study was conducted in accordance with the principles of the Declaration of Helsinki and approved by the Ethics Committee of the Universitat de València Estudi General (UVEG). Clinical data (gender, age, tumor size, tumor location, resection margins, adjuvant treatment, recurrences, metastasis, and survival) were also retrieved from the hospital’s digital clinical record. Follow-up data (recurrence, metastases, and final outcomes) were also collected from the corresponding clinical files. Patients were followed for a median period of 79.6 months, providing robust longitudinal data. Further clinicopathological data can be found in Table 1 from our previously published research on the same SS cohort [11].

### 2.1. Histopathology

All the available H&E slides were examined by four pathologists (E.M.-C., F.G., I.M., and A.L.-B.), all blinded to the clinical data. In cases of disagreement, a consensus was reached on a multiheaded microscope. An SS diagnosis was established according to World Health Organization (WHO) criteria [1].

### 2.2. Immunohistochemical Analysis

Immunohistochemistry staining was carried out on 3–4 μm thick formalin-fixed paraffin-embedded tissue microarrays (TMAs). The markers studied included CD163 (10D6—Biocare, Concord, CA, USA), CD68 (KP1—Dako, Santa Clara, CA, USA), CD3 (Polyclonal Rabbit—Dako), CD8 (C8/144B—Dako), and CD20 (L26—Dako). The extent of positive immunohistochemical staining for all antibodies was scored from 0 to 3 (0 = ≤9, 1 = 10–49, 2 = 50–99, and 3 = ≥100 positive cells per 10 high-power fields). Standard positive and negative controls were used throughout. The scores of all observers were recorded, and discordant cases were evaluated using a multiheaded microscope to achieve a consensus.

**Figure 1:**
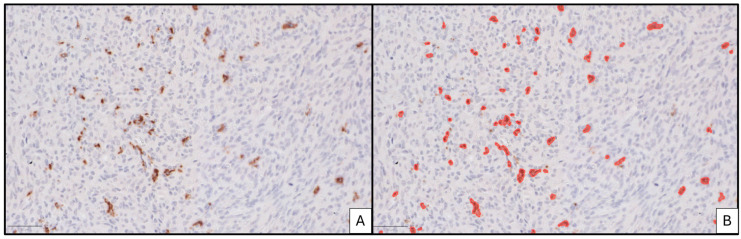
Detection of immune markers using digital pathology. (**A**) CD68-positive macrophages. (**B**) Quantification of the positive area detected through digital analysis.

We repeated the same immunohistochemical panel for the subgroup of 23 cases with available whole-slide tissue and then assessed the positive area by scanning the slides and analyzing the images using QuPath (version 0.4.4, Belfast, Northern Ireland) to calculate the positive area (square microns) in a 2.27 mm^2^ hotspot (equivalent to the area of 10 high-power fields (40×) in conventional light microscopy). The hotspots were manually selected by identifying the intratumoral regions that demonstrated the highest density of inflammatory infiltrate. The selection criteria also included a requirement that the chosen areas were unequivocally located within the tumor boundaries and exhibited adequate histological preservation as well as consistent image quality across all immunohistochemical stains. The positive area was quantified using the cell detection workflow in QuPath, leveraging its pixel classifier tool to differentiate positively stained regions from the background. The classifier was trained based on color deconvolution and relevant pixel intensity features to identify areas of staining. Following the initial classification, the positivity threshold was manually adjusted to refine the detection of truly positive cells, accounting for variations in the staining intensity and background artifacts. This manual calibration ensured the reliability and consistency of the quantification acrossall analyzed samples (Figure 1).

### 2.3. Statistical Analysis

The statistical analysis was conducted using SPSS (v25.0, Armonk, NY, USA) software. The expression of the previously mentioned markers was correlated with clinical outcomes using a log-rank test and Kaplan–Meyer curves as appropriate. For all analyses, *p* < 0.05 was considered statistically significant. The overall follow-up period and the interval until an event (recurrence or metastasis) were calculated from the date of surgical removal or biopsy. The follow-up period for patients who did not experience recurrence was considered as lasting until the date of their most recent clinical check-up or last radiological control.

For survival analysis using digital quantification, expression levels were categorized as “high” or “low” based on the rounded-up median value of each marker.

## 3. Results

This study included 49 fusion-confirmed SS cases from our original cohort of previously published cases, for which we evaluated the histological, immunohistochemical, and molecular findings [11]. Three of these cases included two specimens each, representing both the primary tumor and the resection of the recurrent disease, resulting in a total of 52 tumors being included in the study.

The mean age of our patients was 39.59 years, with a range of between 9 and 82 years. A predominance of males was observed, with 32 male patients compared to 17 females, resulting in a female-to-male ratio of 1:1.8. All SSs in our cohort were classified histologically according to the WHO criteria as monophasic, biphasic, and undifferentiated tumors. The most frequent morphological subtype was monophasic, 31 (59.6%), followed by biphasic, 14 (26.9%), while only 7 (13.5%) were undifferentiated tumors.

Local recurrence was observed in 17 cases (32.69%), and distant metastasis occurred in 24 cases (46.15%), with the lung being the most common metastatic site. Additionally, metastasis was identified in bone (three cases), lymph nodes (two cases), and the costal–pleural region (one case). Eighteen patients died during the follow-up; synovial sarcoma was identified as the direct cause of death in seventeen of these cases. The mean progression-free survival (PFS) was 55.3 months, with a median of 33 months, while the median overall survival (OS) was 50 months, ranging from 2 to 336 months. The mean disease-specific survival (DSS) was also 55.3 months, with a median of 33 months.

As shown in Table 1, conventional immunohistochemical analysis revealed the presence of intratumoral immune cells predominated by CD163+ macrophages, with the exception of two tumors where the TMA was not evaluable. Additionally, a variable proportion of CD68+ macrophages and CD8+ and CD3+ T lymphocytes were observed within the tumor microenvironment (TME). All but two of the tumors showed an absence of intratumoral B lymphocytes. Due to tissue exhaustion in the TMAs, the presence of CD163 could not be adequately evaluated in two cases, and it was therefore excluded from the analysis.

**Figure 2:**
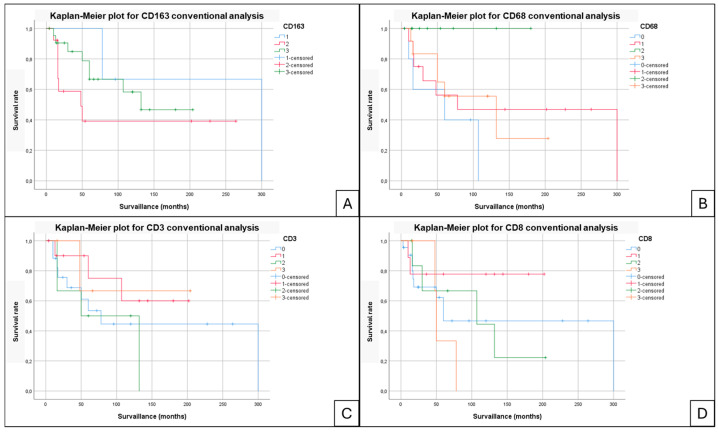
Kaplan–Meier plot curves for conventional TIME immunohistochemical quantification analysis performed in tissue microarrays: (**A**) CD163, (**B**) CD68, (**C**) CD3, and (**D**) CD8.

Survival analysis conducted on TMAs using conventional immunohistochemistry with the three-tier system found no statistical correlations between immune cell infiltrates and the overall survival (Figure 2).

**Figure 3:**
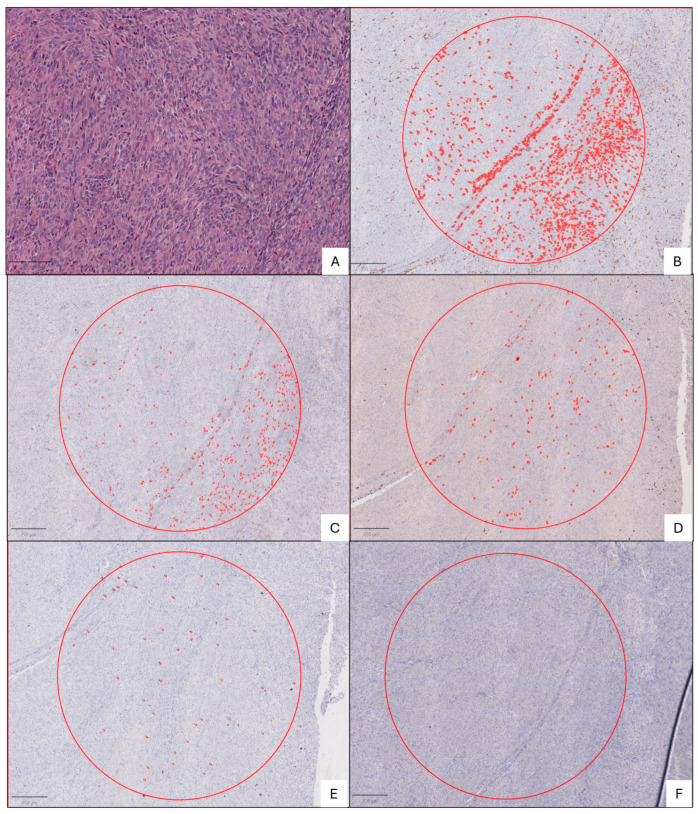
SS digital TME immunohistochemistry analysis. The red circles represent annotations corresponding to a 2.27 mm^2^ area where cell detection was conducted, with detected cells appearing in red. (**A**) H&E monophasic spindle cell SS (tumor 32); (**B**) CD163—39861.8 µ^2^; (**C**) CD68—4531.3 µ^2^; (**D**) CD3—4603 µ^2^; (**E**) CD8—1180 µ^2^; (**F**) CD20—0 µ^2^.

Given the inherent limitations of performing analysis on TMAs, where it is not possible to evaluate the entire tumor and where intratumoral heterogeneity (both neoplastic and inflammatory) is present, a whole-slide study was conducted on tumors with sufficient and suitable material for a better assessment. Tru-cut biopsies, fragmented tumors, and cases in which the material had been extracted from the hospital’s archive for patient care could not be included in the whole-slide analysis. At the time of the analysis, only 23 out of the 52 tumors met this criterion and were therefore analyzed, focusing on intratumoral inflammatory infiltrate hotspots. In these hotspots, the positive staining area for antibodies was quantified across an area equivalent to 10 high-power fields (40× magnification) (Figure 3).

**Figure 4:**
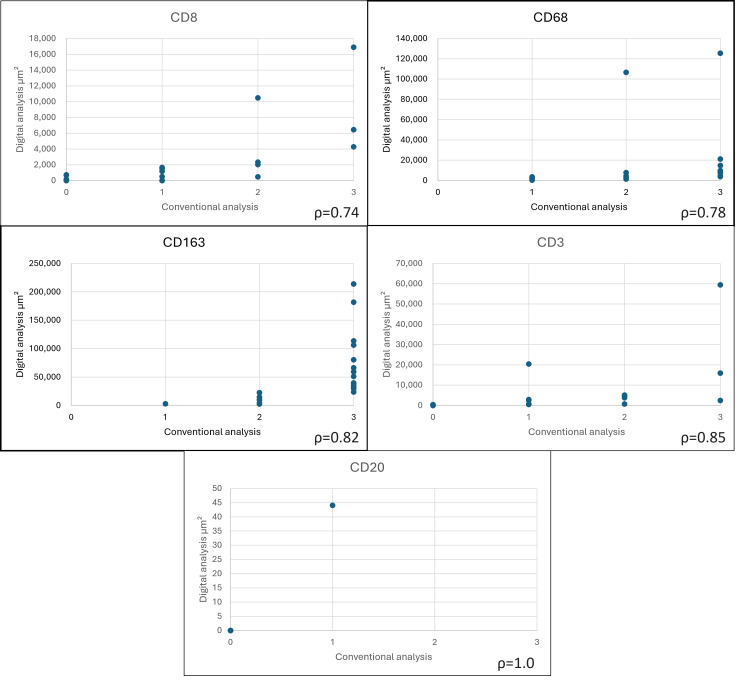
Scatter plots comparing the two immunohistochemical interpretation methods (digital and conventional) to evaluate the differences and correlations between the approaches. Spearman’s ρ values are represented for each marker.

A comparative analysis was performed between the two immunohistochemical interpretation methods (digital and conventional), using scatter plots to illustrate the differences and correlations between the two approaches (Figure 4). The Spearman correlation coefficients indicated a moderate to high correlation for CD3 (ρ = 0.85), CD163 (ρ = 0.82), CD8 (ρ = 0.74), and CD68 (ρ = 0.78). In contrast, due to the absence of B lymphocytes in most cases, the correlation for CD20 was optimal (ρ = 1.0).

**Figure 5:**
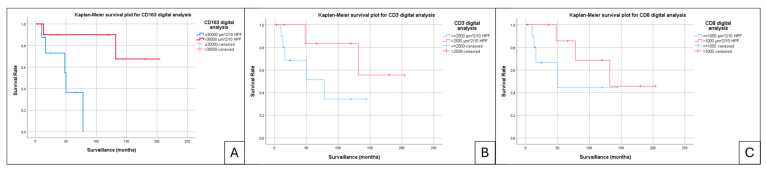
Kaplan–Meier plot curves for digital TIME analysis: (**A**) CD163, (**B**) CD8, and (**C**) CD3.

The dominant intratumoral-infiltrating immune cell type was CD163-positive macrophages, appearing in variable amounts in all cases. The cases with a higher density (>30,000 µm^2^) of CD163-positive macrophages in whole-slide images subjected to digital analysis demonstrated an improved OS and DSS on Kaplan–Meier curves (*p* = 0.013) (Figure 5A) compared to the cases that presented reduced macrophagic infiltrate.

Kaplan–Meier OS analysis revealed non-significant trends associated with the T lymphocytic infiltrate, as measured by CD3 and CD8 markers (Figure 5B,C). Cases with lower CD8 positivity (<1000 µm^2^) and lower CD3 positivity (<2500 µm^2^) showed a tendency toward faster progression and slightly worse outcomes. However, these findings did not reach statistical significance, with *p*-values of 0.165 for CD3 and 0.292 for CD8.

## 4. Discussion

The TME has a key influence on how cancers develop and progress, including for SS and other soft tissue sarcomas. The dynamic interplay between neoplastic cells, immune cells, stromal components such as the stroma and vasculature, and signaling molecules, creates a complex system that influences tumor behavior. Immune cells within the TME, particularly tumor-infiltrating lymphocytes (TILs) and macrophages, exhibit dual roles, acting as both promoters and suppressors of tumor progression. For instance, CD8+ T cells, traditionally regarded as cytotoxic, are often rendered dysfunctional in an immunosuppressive TME, while macrophages shift toward pro-tumorigenic phenotypes under chronic inflammatory conditions [12,13]. This intricate balance between pro- and anti-tumoral forces highlights the importance of studying the TME to understand the mechanisms of immune evasion and resistance to therapy [14,15].

Recent studies have highlighted the involvement of the TME in immune escape and therapeutic response modulation [16,17]. SSs, like other soft tissue tumors, exhibit heterogeneous immune infiltrates, with CD163+ macrophages and regulatory T cells contributing to an immunosuppressive condition. In addition, immune checkpoint molecules such as PD-1/PD-L1 are upregulated in various sarcoma subtypes, further dampening effective immune responses. However, the low mutational burden and limited neoantigen expression characteristic of synovial sarcomas pose challenges to immunotherapy [16,18]. Despite these obstacles, understanding the cellular and molecular dynamics of the TME opens up alternative possibilities for innovative therapeutic strategies, such as targeting immune-modulating pathways or leveraging adaptive cell therapies to enhance anti-tumor immunity.

The TIME of sarcomas is a critical yet underexplored aspect of their biology, contributing to both tumor progression and therapeutic resistance. Soft tissue sarcomas are often classified as “cold” tumors due to their low mutational burden and the limited infiltration of immune cells, including TILs [19,20]. However, studies have shown that even within this relatively immunosuppressive environment, the presence of a high TIL density, particularly of CD8+ cytotoxic T cells, correlates with improved clinical outcomes such as longer disease-free and overall survival [20]. These effects are particularly evident when the TME is reprogrammed using therapeutic strategies such as neoadjuvant chemotherapy combined with regional hyperthermia, which can enhance immune cell infiltration and reduce the level of immunosuppressive factors like FOXP3+ regulatory T cells [20]. Such approaches not only enhance cytotoxic T cell activity but also modulate the TME to favor anti-tumor immunity through mechanisms such as antigen presentation, cytokine production, and the reduced expression of immunosuppressive ligands [21].

Despite these promising findings, the TME in sarcomas remains highly immunosuppressive, characterized by elevated levels of regulatory T cells, macrophages with an M2-like phenotype, and the expression of immune checkpoint molecules such as PD-1 and PD-L1 [22,23]. The upregulation of these immune checkpoint pathways allows tumors to evade immune surveillance, highlighting the need for interventions like checkpoint blockade therapies to restore effective T cell function [20].

Given that sarcomas account for a very small percentage of all malignant tumors, studies focusing on the TME often include multiple sarcoma subtypes and rarely concentrate on a specific type. However, a previous study published by Oike N. et al. [24] explored the TME in SS. The authors reported findings similar to ours with respect to lymphoid and macrophage infiltrates. In their cohort of 36 SS cases, all the tumors exhibited infiltration by CD163+ macrophages, a fact mirrored by the results observed in our study. However, the correlation with the outcomes differed markedly, as the above-mentioned study found that the presence of CD163+ macrophages was associated with improved PFS. This significance level is less robust compared to the stronger correlation observed in our study. The presence of CD163-positive macrophages has been reported as a prognostic marker across various sarcoma subtypes, including leiomyosarcoma, myxoid liposarcoma, undifferentiated pleomorphic sarcoma, embryonal rhabdomyosarcoma, and synovial sarcoma [25,26]. Tumor-infiltrating macrophages play a dual role in the TME, influencing both tumor progression and therapeutic outcomes. CD163+ macrophages, often associated with an M2-like phenotype, contribute to immunosuppression, angiogenesis, and metastasis in various sarcoma subtypes; these macrophages secrete key cytokines, such as IL-6 and CXCL2, which are pivotal in enhancing tumor cell proliferation and survival via pathways such as STAT3 activation. Silencing CD163 in macrophages removes their ability to induce tumor growth, highlighting the receptor’s critical role in macrophage-mediated tumorigenesis [27]. Moreover, CD163 involvement extends beyond cytokine production, as it influences macrophage–tumor cell interactions, facilitating tumor cell adaptation and proliferation, especially during early tumor development [27,28,29]. In leiomyosarcomas, increased tumor-associated macrophage (TAM) infiltration, identified using CD163 and CD68 markers, correlates with worse survival outcomes, particularly in non-gynecologic subtypes, suggesting its utility as a prognostic marker [28,30]. Despite their generally poor prognostic implications, the functional plasticity of TAMs provides an avenue for therapeutic intervention. Evidence from embryonal rhabdomyosarcomas indicates that higher densities of CD163+ macrophages can correlate with better survival, potentially due to their interactions with other immune components, such as CD54+ microvessels, which enhance immune cell recruitment [25].

In our cohort, higher amounts of CD8+ lymphocytic infiltrates were associated with a better OS compared to tumors with scant CD8+ infiltration, a result that aligns with the findings reported by Oike et al. [24]. CD8+ T cells are critical players in anti-tumor immunity, as demonstrated in various tumor models, including sarcomas, where their presence indicates a more immunologically active or “hot” TME. Recent studies in osteosarcoma and other sarcoma subtypes have similarly highlighted a strong correlation between high CD8+ T cell infiltration and improved survival outcomes, emphasizing the significance of these cytotoxic lymphocytes in shaping tumor behavior [31,32]. Single-cell analyses of tumor-infiltrating lymphocytes in soft tissue sarcomas, including Ewing sarcoma and other subtypes, have underscored the important role of CD8+ T cells in anti-tumor immune responses. High densities of these cells are associated with favorable clinical outcomes, supporting their use as potential prognostic markers and therapeutic targets. Although our study did not evaluate PD-L1 expression, findings from other sarcoma studies suggest that CD8+ T cells may exert their effects independently, underscoring their critical role in tumor immune dynamics and therapeutic responsiveness [33,34,35]. However, the interaction between CD163+ macrophages and CD8+ T cells can significantly influence this dynamic. Studies have demonstrated that TAMs, particularly those expressing CD163, can physically impede CD8+ T cells from reaching tumor cells and penetrating the tumor parenchyma, thereby limiting their movement and effectiveness within the tumor stroma. This macrophage-mediated restriction contributes to immune exclusion, where CD8+ T cells remain confined to the tumor stroma, reducing their capacity for direct cytotoxic interactions with malignant cells [36].

Digital pathology has allowed for the analysis of TILs and TAMs, as highlighted in our study, where whole-slide digital image analysis proved superior to conventional interpretation. While TMAs provide limited sampling and often fail to capture the full heterogeneity of immune cell distribution, whole-slide imaging enables the precise identification and quantification of hotspots, ensuring a comprehensive evaluation of immunomarkers across the heterogeneity of a tumor and enabling the detailed quantification of areas of a high immune cell density [37,38]. This approach aligns with broader advancements in computational pathology, where algorithms not only enhance accuracy but also provide reproducibility [39,40]. Furthermore, incorporating spatial metrics into digital analyses offers critical insights into the TME, including the proximity and interaction patterns between immune and tumor cells, which are often overlooked in conventional analysis and interpretation [41,42].

The main limitation of our study was the lack of access to whole-slide imaging for all the cases. However, even with a more limited sample, important data were obtained that conventional analysis could not generate. Additionally, the quality of the digitized images was not optimal, highlighting the potential of newer technologies with a higher resolution to enhance analysis. Furthermore, we did utilize manual adjustments to the cell detection thresholds, which could have generated some bias. Also, we did not utilize artificial intelligence algorithms for detecting positive cells, which presents an opportunity to improve the reproducibility and precision of future analyses by integrating artificial intelligence tools into the analysis. Lastly, our immunohistochemical profile was limited and could be expanded to include additional markers, such as FOXP3, to provide a more comprehensive characterization of the TIME. We acknowledge that the lack of multivariate Cox analysis limited the ability to confirm the independent prognostic value of immune markers, but the small sample prevented the development of a reliable multivariable model.

## 5. Conclusions

The TIME in sarcomas is a complex system that requires further investigation to fully understand its impact on tumorigenesis and patient clinical outcomes. Our results demonstrate that a higher amount of intratumoral CD163+ macrophage infiltrate is associated with an increased OS and DSS. Additionally, we observed trends suggesting that higher CD8+ and CD3+ T cell densities are associated with better clinical outcomes, underscoring the potential role of adaptive immune responses in synovial sarcoma. These findings were obtained using digital pathology, which proved to be a superior tool compared to subjective quantitative analysis by enabling the precise measurement of immune cell infiltrates and reducing observer bias.

## Figures and Tables

**Table 1:** Summary of the conventional immunohistochemical analysis of 52 synovial sarcoma cases, evaluating the expression of CD163, CD68, CD8, CD20, and CD3. Three-tier score system: 0 = ≤9, 1 = 10–49, 2 = 50–99, and 3 = ≥100 positive lymphocytes or macrophages in 10 high-power fields.

Score	CD163	CD68	CD8	CD20	CD3
0	0	6	25	50	20
1	3	18	11	1	18
2	17	13	9	0	8
3	30	15	7	1	6
Total cases	50	52	52	52	52

## Data Availability

The original contributions presented in the study are included in the article; further inquiries can be directed to the corresponding author.
